# Estimating the number of colorectal cancer patients treated with anti-tumour therapy in 2015: the analysis of the Czech National Cancer Registry

**DOI:** 10.1186/1471-2458-12-117

**Published:** 2012-02-10

**Authors:** Tomáš Pavlík, Ondřej Májek, Jan Mužík, Jana Koptíková, Lubomír Slavíček, Jindřich Fínek, David Feltl, Rostislav Vyzula, Ladislav Dušek

**Affiliations:** 1 Institute of Biostatistics and Analyses, Masaryk University, Brno, Czech Republic; 2 Department of Oncology, Hospital in Jihlava, Jihlava, Czech Republic; 3 Oncology and Radio-therapeutic Department, University Hospital, Plzen, Czech Republic; 4 Clinic of Oncology, University Hospital in Ostrava, Ostrava, Czech Republic; 5 Department of Complex Oncology Care, Masaryk Memorial Cancer Institute, Brno, Czech Republic

## Abstract

**Background:**

Colorectal cancer (CRC) represents a serious health care problem in the Czech Republic, introducing a need for a prospective modelling of the incidence and prevalence rates. The prevalence of patients requiring anti-tumour therapy is also of great importance, as it is directly associated with planning of health care resources.

**Methods:**

This work proposes a population-based model for the estimation of stage-specific prevalence of CRC patients who will require active anti-tumour therapy in a given year. Its applicability is documented on records of the Czech National Cancer Registry (CNCR), which is used to estimate the number of patients potentially treated with anti-tumour therapy in the Czech Republic in 2015.

**Results:**

Several scenarios are adopted to cover the plausible development of the incidence and survival rates, and the probability of an anti-tumour therapy initiation. Based on the scenarios, the model predicts an increase in CRC prevalence from 13% to 30% in comparison with the situation in 2008. Moreover, the model predicts that 10,074 to 11,440 CRC patients will be indicated for anti-tumour therapy in the Czech Republic in 2015. Considering all patients with terminal cancer recurrence and all patients primarily diagnosed in stage IV, it is predicted that 3,485 to 4,469 CRC patients will be treated for the metastatic disease in 2015, which accounts for more than one third (34-40%) of all CRC patients treated this year.

**Conclusions:**

A new model for the estimation of the number of CRC patients requiring active anti-tumour therapy is proposed in this paper. The model respects the clinical stage as the primary stratification factor and utilizes solely the population-based cancer registry data. Thus, no specific hospital data records are needed in the proposed approach. Regarding the short-term prediction of the CRC burden in the Czech Republic, the model confirms a continuous increase in the burden that must be accounted for in the future planning of health care in the Czech Republic.

## Background

The Czech population, with an annually diagnosed 78.7 colorectal cancer (CRC) patients per 100,000 inhabitants (2008), presently occupies an undesirable 3rd position in international statistics of age-standardised CRC incidence rates [1]. Moreover, the number of newly diagnosed cases is supposed to be high in the future as well, namely due to population ageing. This health care problem is further worsened by the fact that a large proportion of colorectal carcinomas are primarily diagnosed in a metastatic stage (25% in 2008) [2].

Thus, there is a strong need for a prospective modelling of CRC incidence and prevalence rates, as these measures are necessary for monitoring of the overall cancer load and its dynamics [3]. The prospective estimates should also enable us to quantify the resources necessary for the health care system [4], provided that we are able to adjust the rates for patients untreated for whatever reason (treatment contraindication, patient's refusal of treatment, very advanced stage of disease or unfavourable health status of a patient, etc.). The existing models use either only population data [5]or a combination of population data and clinical records [6,7]. In the former case, the model does not employ a concept of cancer recurrence, whereas in the latter case, the concept of cancer recurrence is considered and the particular rates are estimated from the hospital records.

The objective of this paper is to propose a new model for the estimation of the number of CRC patients requiring active anti-tumour therapy that is fully based on the population-based cancer registry data. The model uses the population data for identification of all its components, i.e. also for the quantification of cancer recurrence rates. Moreover, the proposed model is stage-specific and as such it respects the disease extent at the time of primary diagnosis. The stage-specific estimates of the CRC burden can be regarded as the main added value of this paper. This paper does not focus on the specific anti-tumour therapies as well as their combinations. The reason for this is that the use of population data is not sufficient for proper characterisation and quantification of the individual treatment modalities. Thus, the anti-tumour therapy is considered rather as a whole. On the other hand, the information on use of the individual treatment modalities is partially included within the information on stage-specific CRC burden as the clinical stage closely correlates with particular therapeutic procedures. The model is finally applied to the Czech population data and provides the estimates of CRC patients treated in the Czech Republic in 2015.

## Methods

### Patients

The Czech Republic makes use of high-quality population-based data on cancer epidemiology. The Czech National Cancer Registry (CNCR) covers the whole population of the Czech Republic (10,230,000 inhabitants according to the 2001 census) since 1976. The registration of cancer cases is prescribed by law and it is therefore obligatory. Until the end of 2008, there were almost 1.7 million cancer cases recorded in the CNCR. A total of 179,286 incident CRC cases (12% of the CNCR records) were registered in the period 1982-2008. Cancer location was defined according to the International Classification of Diseases [8]. ICD-10 codes C18, C19, and C20 were considered as CRC cases. Regarding only CRC as the cancer of interest, the completeness of CRC prevalence in the CNCR can be estimated, using the model-based method utilising the observed and modelled CRC prevalence [9], to be approximately 97% in 2008. The CNCR records on cancer as the main cause of death were verified against the Czech Database of Death Records, a database administered by the Czech Statistical Office [10].

Only clinically relevant cancer records entered the modelling procedures. Data on cases diagnosed in 1977-1981 were excluded due to the lack of a classification system for clinical stages. The epidemiological records on patients diagnosed by death certificate only (3,943 cases in total, 2% of all incident CRC cases) or at autopsy (7,184 cases in total, 4% of all incident CRC cases) were excluded from the analysis as well. Finally, 160,017 incident cases were considered for the analysis, 38% younger than 65 years, and 57% males.

Four age categories were considered in the modelling: 0-49 years, 50-64 years, 65-79 years and 80+ years; as well as three categories for the disease extent: clinical stages I and II, representing localised CRC, clinical stage III, representing regionally advanced disease, and clinical stage IV, representing metastatic disease. Colorectal cancers in stages I and II were merged prior to analyses due to changes in the TNM classification system [11]. Moreover, cases with missing information on stage (denoted as X) were also considered for the model, as they represent an indispensable mass of patients that needs to be accounted for in the health care system.

Active anti-tumour therapy was defined on the basis of the CNCR records as causal therapy; symptomatic therapy was not considered. This definition includes surgery, radiotherapy, chemotherapy, hormonal therapy, targeted therapy, and immunotherapy [12].

### The proposed model concept

The concept comes from the model of time interval prevalence, a number of patients who both have a present or past diagnosis of CRC and are alive in a population during a certain period. In this part of the paper, for the sake of formula simplicity, we consider only the case of one particular age group. The overall number of prevalent cases across all age groups can easily be estimated as a sum of predictions over individual age categories. Regarding the extent of cancer, denoted with *s *(*s *= I + II, III, IV and X), the stage-specific prevalence in calendar year *y *can be expressed as follows:

(1) $$P_{s}\left(y\right)=I_{s}\left(y\right)+\overset{n}{\underset{i=1}{\sum }}I_{s}\left(y-i\right)S_{s}\left(i\right).$$

Here *I_s_*(*y - i*) denotes the stage-specific incidence at *i *years prior to the calendar year *y, S_s_*(*i*) is the corresponding *i-*year survival rate, and *n *is the maximum follow-up in the population-based registry in years (given either by the registration period or by the modelling process). Note that the first term on the right-hand side of equation (1) represents the number of newly diagnosed patients in year *y*, whereas the second term stands for the number of living patients diagnosed in the past, which is estimated as the convolution of the incidence rates and the appropriate patients' survival rates.

To obtain the number of treated patients, the first term of equation (1) needs to be further corrected for the probability of being untreated with anti-tumour therapy due to poor health condition or other objective reasons, and, simultaneously, the second term of equation (1) needs to be corrected in a way that the only patients considered are those with the recurrence of the disease in a good health condition to allow for anti-tumour treatment. The prevalence of patients receiving active anti-tumour therapy can then be written as follows:

(2) $$P_{s}^{*}\left(y\right)=I_{s}\left(y\right)\delta_{s}\left(y\right)+\overset{n}{\underset{i=1}{\sum }}I_{s}\left(y-i\right)S_{s}\left(i\right)R_{s}\left(i\right)\delta_{s}\left(y\right).$$

Here *δ_s_*(*y*) is the stage-specific probability of being treated with an anti-tumour treatment in the year *y *and *R_s_*(*i*) is a function that describes the probability of cancer recurrence after surviving *i *years after primary diagnosis.

The cancer recurrence function, *R_s_*(*i*), is further specified using the following consideration: each patient diagnosed in stage *s *can suffer in time from two forms of cancer recurrence, either non-terminal (actually not leading to death in the year *y*, denoted as *R_s_*^1^(*i*)), or terminal (leading to death in the year *y*, denoted as *R_s_*^2^(*i*)). The stratification further determines the patient's treatment course. In the former case, it is assumed that the patient is treated in a similar way as at the time of primary diagnosis, i.e. the patient stays in the prevalence pool of the particular stage *s*. In the second case, it is assumed that the patient is treated for metastatic disease, i.e. the patient moves from the prevalence of stage I + II, III or X to the prevalence of stage IV.

Splitting the *R_s_*(*i*) term in equation (2), and moving the patients suffering from terminal cancer recurrence to the prevalence of stage IV, led to the following formulation of the stage-specific prevalence of patients requiring active anti-tumour therapy:

(3) $$P_{s}^{*}\left(y\right)=I_{s}\left(y\right)\delta_{s}\left(y\right)+\overset{n}{\underset{i=1}{\sum }}I_{s}\left(y-i\right)S_{s}\left(i\right)R_{s}^{1}\left(i\right)\delta_{s}\left(y\right);\;s=\text{I}+\text{II,III,X}$$

(4) $$\begin{array}{c} P_{\text{IV}}^{*}\left(y\right)=I_{\text{IV}}\left(y\right)\delta_{\text{IV}}\left(y\right)+\overset{n}{\underset{i=1}{\sum }}I_{\text{IV}}\left(y-i\right)S_{\text{IV}}\left(i\right)\left(R_{\text{IV}}^{1}\left(i\right)+R_{\text{IV}}^{2}\left(i\right)\right)\delta_{\text{IV}}\left(y\right)\\ +\underset{s=\text{I}+\text{II,III,X}}{\sum }\overset{n}{\underset{i=1}{\sum }}I_{s}\left(y-i\right)S_{s}\left(i\right)R_{s}^{2}\left(i\right)\delta_{\text{IV}}\left(y\right). \end{array}$$

It should be noted that the probability of being treated is assumed to be the same in all patients primarily diagnosed with stage IV cancer, denoted as *δ*_IV_(*y*), irrespective of whether they are newly diagnosed or suffer from non-terminal or terminal cancer recurrence, respectively. On the other hand, in patients primarily diagnosed with stage *s *= I + II, III or X, the stage-specific probability of being treated is assumed to be the same, and denoted as *δ_s_*(*y*), only in newly diagnosed patients and those patients suffering from non-terminal cancer recurrence. In patients estimated to suffer from terminal cancer recurrence who are supposed to move from the prevalence of stage I + II, III or X to the prevalence of stage IV, we assume the probability of being treated to be equal to that of patients primarily diagnosed with stage IV cancer, i.e. equal to *δ*_IV_(*y*).

### Specifying colorectal cancer incidence and survival

Two scenarios are considered for the estimation of incidence rates. First, CRC incidence rates are considered fixed at the values observed in 2008; second, the age, period and cohort model is applied for the estimation [13]. In the proposed model, the age-drift Poisson regression models are applied employing two different link functions: the identity link function is used to model increasing incidence trends, whereas the logarithmic link is used to model decreasing trends [14].

The stage-specific survival rates are estimated using a method based on the moving window principle that employs the standard life-table method [15]. In this procedure, the survival rates are estimated successively, using the cohort analysis of patients diagnosed in overlapping 5-year time intervals. To ensure validity, calculation of *x*-year survival rates is only performed on cohorts in whom the *x*-year survival rate can be reliably estimated, and were diagnosed as recently as possible [16]. Two scenarios are adopted regarding survival estimates. In the first scenario, the survival rates are assumed to improve from 2008 to 2015 in the same manner as observed in the CNCR data from the period of 2004-2008. In the second scenario, survival rates needed for calculating the 2009-2015 prevalence are fixed at the most recent values, i.e. survival rates in 2008.

### Colorectal cancer recurrence

The records of cancer recurrence rates are not directly available on the population level in the Czech Republic. For this reason, we use surrogate parameters to estimate the cancer recurrence rates. Regarding non-terminal cancer recurrence, *R_s_*^1^(*i*), it is estimated using the follow-up information on the patient's vital status (recorded as alive or dead) and the information on causal anti-tumour therapy applied in yearly intervals after the first year following diagnosis (the very first year after diagnosis is assumed to correspond to the initial treatment phase [17]). This information is extracted from the CNCR Follow-up Reports that represent obligatory monitoring of cancer patients in the Czech Republic in time. We simply assume that a record on other than symptomatic therapy in a particular year after diagnosis indicates that the patient is treated in this year due to an objective reason, i.e. due to the return of cancer. However, as the *R_s_*^1^(*i*) function refers only to non-terminal cancer recurrence, an additional condition is necessary, namely that the patients must be alive at the end of the particular year of interest, i.e. the cancer recurrence must not be terminal in a given year. This condition is applied to ensure that the patient is not in a terminal (metastatic) stage of CRC in the year of interest. The *R_s_*^1^(*i*) function is estimated from the yearly Follow-up Reports using the standard life-table method.

As for terminal cancer recurrence, the *R_s_*^2^(*i*) function is estimated using the information on cancer as the main cause of death. The approach is based on the simplifying assumption that nobody can die from cancer, with cancer being the main reason of death, without passing through the phase of metastatic disease. In other words, we assume that even a patient diagnosed with stage I CRC is treated as metastatic (stage IV) in a given year when cancer is recorded as his cause of death. The *R_s_*^2^(*i*) function thus represents the excess mortality of the colorectal cancer, i.e. the difference between the total mortality experienced by the CRC patients and the expected mortality of a comparable group from the general population, and can be thus specified using either the relative survival function or the underlying excess hazard rate [18]. Both of these quantities are derived using the mixture cure survival model adjusted for background mortality [19].

As the last factor needed for the model, the age- and stage-specific proportions of patients treated with anti-tumour therapy are derived from the CNCR population data. Just as in the case of incidence and survival rates, two scenarios are considered for the proportion of the treated CRC patients. First, this proportion is regarded as fixed and estimated in a stage-specific manner from the period 2004-2008. Second, the values observed from the CNCR are extrapolated forward in time using a logistic regression model.

For the sake of completeness, the eight models considered in this paper that are defined by combination of two scenarios for the estimation of incidence rates (fixed and modelled, respectively), two scenarios for the estimation of survival rates (constant and improving, respectively), and two scenarios for the proportion of treated patients (fixed and modelled, respectively) are summarised in Table 1. All computations were performed using Stata 10.1 software [20].

**Table 1:** Description of the eight scenarios used to estimate the number of colorectal cancer patients treated with anti-tumour therapy in 2015 in the Czech Republic

Proportion of treated patients (for the year 2015)	Incidence rates (for the period 2009-2015)	Survival rates (for the period 2009-2015)	
		
		Survival rates are considered fixed at the most recent values, i.e. survival rates in 2008	Survival rates are assumed to improve in the same manner as observed in the period 2004-2008
Proportion is regarded fixed in time and estimated from the period 2004-2008	Incidence rates are considered fixed at the values observed in 2008	*Scenario 1*	*Scenario 2*
	
	Incidence rates are modelled in time using the age-drift Poisson regression model	*Scenario 3*	*Scenario 4*

Proportion observed in the period 2004-2008 is extrapolated forward in time using a logistic regression model	Incidence rates are considered fixed at the values observed in 2008	*Scenario 5*	*Scenario 6*

	Incidence rates are modelled in time using the age-drift Poisson regression model	*Scenario 7*	*Scenario 8 *

## Results

**Figure 1:**
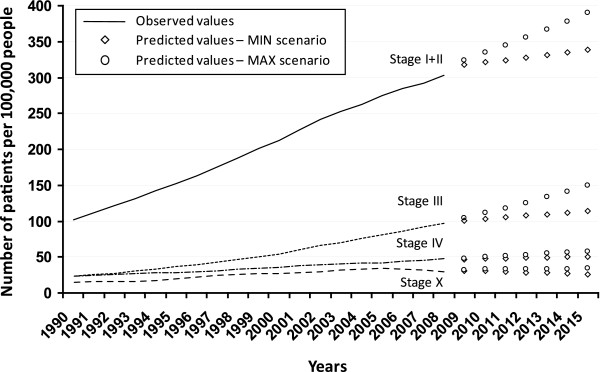
**Observed and predicted values of colorectal cancer prevalence in the Czech Republic per 100,000 people according to clinical stage of primary tumour**.

Figure 1 shows the observed and predicted values of one-year CRC prevalence in the Czech Republic. In order to simplify the results, only the lowest and the highest estimates coming from the four scenarios that reflect different trends in incidence and survival rates are displayed. The lowest estimates have been obtained for all stages when applying the scenarios with constant survival rates. The highest estimates for stages I + II and patients with missing information on stage have been obtained by applying the scenario with constant incidence and improving survival rates, whereas the highest estimates for stage III and stage IV have been obtained by applying the scenario with modelled incidence and improving survival rates. As expected, the biggest discrepancy between the scenarios can be seen for the merged stages I + II where the improvements in survival are manifested the most. In 2015, the CRC prevalence of patients primarily diagnosed in stage I or II is estimated as ranging between 338.8 and 389.8 per 100,000 people, while the prevalence of patients diagnosed in stage III is estimated as ranging between 114.1 and 150.2 per 100,000 people, and the prevalence of patients diagnosed in stage IV ranging between 50.7 and 58.1 per 100,000 people. The prevalence of CRC patients with missing information on stage in CNCR is estimated as ranging between 26.3 and 33.9 per 100,000 people. In total, between 529.9 and 632.0 CRC patients per 100,000 people are estimated to be prevalent in 2015. The model thus predicts an increase in CRC prevalence from 13% to 30% in comparison with the situation in 2008. This increase underlines the seriousness of the colorectal cancer burden in the Czech Republic.

**Figure 2:**
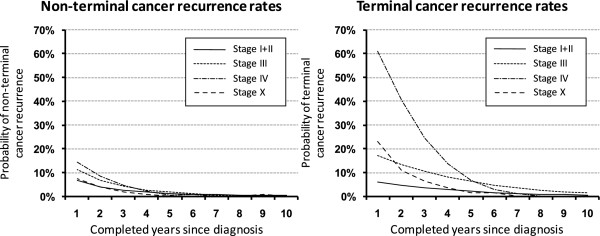
**Stage-specific estimates of non-terminal and terminal recurrence rates in first ten years after primary diagnosis of colorectal cancer; the estimates correspond to the recent time period, 1995-2008**.

Figure 2 shows the estimated stage-specific rates of non-terminal and terminal cancer recurrence, respectively; in the ten years following the first completed year after diagnosis (the first year after diagnosis is considered to correspond to primary therapy). The estimates corresponding to the most recent time period, 1995-2008, are shown. We can see the risk of non-terminal cancer recurrence gradually decreasing in the first three years and reaching the 3% level in all stages afterwards. On the contrary, the pattern of terminal recurrence rates varies with clinical stage up to five years after diagnosis; in stages I + II, the recurrence rates are consistently below 7%, conveying a good perspective of patients diagnosed with less advanced disease. In stage III, the terminal recurrence rate shows a stable but very slow decrease in time. The terminal recurrence rate for stage IV reveals a very high risk of dying from CRC exceeding even 60% after the first year following diagnosis. The risk reaches the level comparable to other stages after 5-6 years following the diagnosis. The terminal recurrence rate of the patients with missing information on stage is located in the middle of the other stage-specific profiles (Figure 2). It documents the fact that patients with missing information on stage represent a mixture of patients of all stages.

Based on CNCR data, the proportion of patients who are given non-symptomatic anti-tumour treatment in stages I + II and III is continually high (above 95%) and stable in time (data not shown). The only exception is the group of elderly patients (80+ years), where the proportion of treated patients is approximately 85%. On the other hand, we can see a change in the probability of an anti-tumour therapy administration in patients treated for CRC in stage IV. We have estimated the shift in the proportion of treated stage IV patients between the period 2004-2008 and the year 2015 to be from 3% (a change from 90% in the period 2004-2008 to 93% estimated for the year 2015) in the age group 0-49 years up to 18% (a change from 44% to 62%) in the age group 80+ years.

The numbers of patients requiring active anti-tumour therapy for the CRC in the Czech Republic in 2015 estimated according to eight considered scenarios are given in Table 2. For each scenario, the first three columns represent the individual components of the proposed model: the estimated number of newly diagnosed and treated patients, the estimated number of patients treated for non-terminal cancer recurrence, and the estimated number of patients treated for terminal cancer recurrence, respectively. Then, the sums with respect to the stage at the diagnosis are shown (column 4).

**Table 2:** Stage-specific estimates of prevalence of patients requiring active anti-tumour therapy for colorectal cancer in the Czech Republic in 2015 according to the eight scenarios

	Scenario 1: Constant proportion of treated patients; Constant incidence rate; Constant survival rates				Scenario 2: Constant proportion of treated patients; Constant incidence rate; Improving survival rates			
Stage at diagnosis	Newly diagnosed patients	Non-terminal cancer recurrence	Terminal cancer recurrence	Total	Newly diagnosed patients	Non-terminal cancer recurrence	Terminal cancer recurrence	Total
Stage I+II	3,650	565	479	4,694	3,650	607	524	4,781
Stage III	1,783	355	541	2,679	1,783	407	625	2,815
Stage IV	1,419	181	766	2,366	1,419	212	898	2,529
Missing	220	16	99	335	220	13	76	309

All cases	7,072	1,117	1,885	10,074	7,072	1,239	2,123	10,434

	Scenario 3: Constant proportion of treated patients; Modelled incidence rate; Constant survival rates				Scenario 4: Constant proportion of treated patients; Modelled incidence rate; Improving survival rates			

Stage at diagnosis	Newly diagnosed patients	Non-terminal cancer recurrence	Terminal cancer recurrence	Total	Newly diagnosed patients	Non-terminal cancer recurrence	Terminal cancer recurrence	Total
Stage I+II	3,581	547	467	4,595	3,581	589	511	4,681
Stage III	2,223	422	632	3,277	2,223	475	725	3,423
Stage IV	1,428	177	761	2,366	1,428	207	892	2,527
Missing	131	10	59	200	131	9	47	187

All cases	7,363	1,156	1,919	10,438	7,363	1,280	2,175	10,818

	Scenario 5: Modelled proportion of treated patients; Constant incidence rate; Constant survival rates				Scenario 6: Modelled proportion of treated patients; Constant incidence rate; Improving survival rates			

Stage at diagnosis	Newly diagnosed patients	Non-terminal cancer recurrence	Terminal cancer recurrence	Total	Newly diagnosed patients	Non-terminal cancer recurrence	Terminal cancer recurrence	Total
Stage I+II	3,613	560	562	4,735	3,613	602	613	4,828
Stage III	1,831	362	628	2,821	1,831	415	727	2,973
Stage IV	1,675	206	890	2,771	1,675	241	1,038	2,954
Missing	122	9	121	252	122	7	92	221

All cases	7,241	1,137	2,201	10,579	7,241	1,265	2,470	10,976

	Scenario 7: Modelled proportion of treated patients; Modelled incidence rate; Constant survival rates				Scenario 8: Modelled proportion of treated patients; Modelled incidence rate; Improving survival rates			

Stage at diagnosis	Newly diagnosed patients	Non-terminal cancer recurrence	Terminal cancer recurrence	Total	Newly diagnosed patients	Non-terminal cancer recurrence	Terminal cancer recurrence	Total
Stage I+II	3,542	544	549	4,635	3,542	583	599	4,724
Stage III	2,285	429	734	3,448	2,285	484	844	3,613
Stage IV	1,697	203	887	2,787	1,697	236	1,036	2,969
Missing	72	5	71	148	72	5	57	134
All cases	7,596	1,181	2,241	11,018	7,596	1,308	2,536	11,440

In total, from 10,074 to 11,440 CRC patients are predicted for anti-tumour therapy administration in the Czech Republic in 2015 according to the eight scenarios considered for incidence and survival rates and the probability of anti-tumour therapy administration. When regarding the stage at diagnosis as the primary stratification factor, 4,595 to 4,828 patients (41-47% of all CRC patients) primarily diagnosed in stage I or II; 2,679 to 3,613 patients (27-32%) primarily diagnosed in stage III; and 2,366 to 2,969 patients (23-27%) primarily diagnosed in stage IV are estimated to be treated in 2015, respectively. Regarding patients with missing information on stage, 134 to 335 of them (1-3%) are predicted for anti-tumour therapy in 2015.

Regarding the overall number of patients treated in stage IV in 2015, the patients with terminal cancer recurrence primarily diagnosed in stages I, II or III and the patients with missing information on stage also need to be added to the number of patients primarily diagnosed in stage IV, because they are treated for the metastatic disease, as well. In this case, 3,485 to 4,469 CRC patients are predicted to be treated for the metastatic disease, which accounts for more than one third (34-40%) of all CRC patients that are estimated to be treated in 2015 in the Czech Republic.

## Discussion

Modelling the prevalence of the CRC patients requiring active anti-tumour therapy is an important issue [4-7], especially in countries like the Czech Republic which ranks among countries with the highest cancer load worldwide [1]. Moreover, the effort to estimate the prevalence on a stage-specific basis is a challenging task and, to our knowledge, there is relatively little information on this subject in the literature [21]. The stage-specific modelling is complicated and requires a comprehensive approach, since the stage at the time of diagnosis need not necessarily coincide with the disease extent several years afterwards. The disease extent should be taken into account in the modelling process at all time points because the clinical stage is, in regards to patient life-expectation and anticipated financial costs, even more important than age at diagnosis [22]. That is why we attempt to propose a comprehensive statistical method here that may provide such estimates in a stage-specific manner utilizing solely the population-based cancer registry data.

The cancer prevalence estimation is not straightforward, as it cannot be estimated directly from the population-based data due to time limited registration, and thus it has to be modelled. Several methods have been proposed for estimating the future cancer burden based on different modelling strategies, of which the back-calculation method, combining parametric estimates of incidence and survival, is the most frequently used [23,24]. Other approaches include the calculation of individual likelihoods of living with cancer [25], the application of the Markov model [26] or the application of the Bayesian model [27]. The generalization of the completeness index method first introduced by Capocaccia & De Angelis [9] has also been applied [28]. In our model, we also use the back-calculation method. The incidence rates are estimated using an age-drift model [13,29] whereas the survival rates are estimated using a modification of the standard life-table method [16].

In accordance with other epidemiological studies, for example [23], four extreme scenarios regarding progress in incidence and survival rates are implemented to model the CRC prevalence in this paper. The incidence rates are either assumed fixed at the 2008 level or modelled using the age, period, and cohort model. As for the survival rates, they are either assumed to improve from 2008 to 2015 at the same rate as observed in the period of 2004-2008 or fixed at the most recent values, i.e. the survival rates available in 2008.

The impact of the different scenarios on the CRC prevalence is the most remarkable in stages I + II and III, and almost negligible in stage IV. Considering the incidence profiles of individual CRC stages [2], we can say that improving survival rates also play a crucial role in driving CRC prevalence. The results document the improvements in cancer survival of less advanced CRC that have been achieved in the Czech Republic in the last decade [30]. On the other hand, it also documents the fact that the treatment of CRC in clinical stage IV continues to present a formidable challenge.

The estimated one-year prevalence rates are not directly comparable with the international data coming from comparative studies such as [31], since these studies focus primarily on the point prevalence. However, at least a crude comparison shows that the prevalence of CRC in the Czech Republic gradually reaches the situation in the Western and Northern European countries. A very high incidence rate and the already mentioned successively improving survival rates can be regarded as the two main drivers.

The issue of stage-specific estimation of CRC prevalence can be considered controversial due to the non-trivial association between the stage at diagnosis and the gradual progress of cancer during the follow-up period. Cancer recurrence rates of patients diagnosed in the past and living in the year of interest are thus by all means the most appealing and most arguable components of the model. Our simplifying assumption of two forms of cancer recurrence is motivated by the financial aspects of cancer care. The separation of patients with terminal cancer recurrence is needed as the treatment of metastatic disease is significantly more costly than the treatment of non-terminal disease [22].

Two principal types of estimates for the cancer recurrence rates are widely used, either estimates based on clinical or hospital data [32-35] or estimates coming from population-based databases [36,37]. We feel that the estimates coming from the population-based databases may be more appropriate in this type of modelling, as the estimates calculated from hospital data can lead to biased results due to non-representativeness of the underlying set of patients. On the other hand, the precise information on time of cancer recurrence is barely available in the population-based cancer registries. In our model, the rationale behind the estimation of functions representing the non-terminal and terminal cancer recurrence rates, respectively, is to use surrogate parameters. The terminal form of cancer recurrence is estimated from the information on cancer as the main cause of death, whereas the non-terminal form is identified from the information on patient's vital status and anti-tumour therapy applied during the follow-up period. The need for the surrogate information introduces high requirements on the data quality of the population-based registry. A possible problem with the terminal cancer recurrence rate estimation can be seen in patients whose CNCR record does not include cancer as the main cause of death, but who, in fact, died of cancer. Such patients would cause underestimation of the true terminal cancer recurrence rates; however, this problem is marginal in the CNCR data for the reasons given below. The information on cancer as the main cause of death, that forms the basis for the terminal cancer recurrence rate estimation, is being verified against the Czech Database of Death Records and as such can be regarded as highly reliable [38]. Moreover, there are standardised procedures for control of the CNCR records against the health care documentation implemented in both the central and regional data management system of the CNCR [12].

Regarding the CNCR Follow-up Reports that form the basis for the non-terminal cancer recurrence rate estimation, the main problem is a non-negligible proportion of incomplete records with missing information on the applied anti-tumour therapy. In total, 16% of all CRC patients included in this study have missing information on their treatment after the primary therapy. Indeed, this proportion varies with stage and age when it ranges from 6% in elderly patients diagnosed with stage IV CRC to 22% in patients aged 50-64 years and diagnosed with stage I or II CRC. This fact may lead to underestimation of the true non-terminal recurrence rate as we can assume that some of the patients with incomplete Follow-up Reports were, in fact, treated. From this point of view, the number of patients estimated to suffer from the non-terminal CRC recurrence presented in this paper can be regarded as a lower bound of the true number of patients that will have to be treated in the future. On the other hand, this problem concerns only the population data and not the proposed methodology. The cancer recurrence rates can be provided to the model from any other source, for example from hospital data.

Our estimates of colorectal cancer recurrence rates are incomparable with studies that have presented data on cancer recurrence together for all stages [5,7] due to the unknown distribution of individual stages in these studies. On the other hand, our results are fully comparable with the cumulative recurrence rates published for colorectal cancer stages I-III in the study of Manfredi et al. [37]. Our results are concordant with this study, when considering the cancer recurrence in a form of distant metastases. However, regarding 5-year local recurrence rates, our recurrence estimates are higher than those published by Manfredi and colleagues. This can be explained by two factors. First, the differences in the Czech and French health care systems may play a role as well as different patient and tumour characteristics. Second, the use of surrogate information for the identification of non-terminal recurrence rates may influence the results, because this information may not fully mirror the patient's true health status. Nevertheless, our results show that there is a non-zero risk of cancer recurrence even five years after diagnosis, i.e. after the period that has been previously considered as a minimum time for the so-called statistical cure of colorectal cancer [39]. This finding was also reported for rectal cancer [33]. However, future verification on a population-based or hospital-based level with sufficiently long-term follow-up would be of great value.

Considering the most recent changes in CRC epidemiology and care in the Czech Republic, we feel that the most likely scenario for the year 2015 is the one with stabilised incidence rates, improving survival rates, and an increasing proportion of treated patients (see Table 2, scenario 6). The stabilised incidence rates can be expected due to the increase in attendance at the national organised screening program that has been observed during very recent years in the Czech Republic [40]. In addition, both the improvement in survival rates and the increasing proportion of treated patients can be attributed to the establishment of a network of highly specialised Cancer Centres that took place in the Czech Republic in 2006 [41], and the introduction of molecular targeted therapy in recent years.

## Conclusions

A new model for the estimation of the number of CRC patients requiring active anti-tumour therapy in a stage-specific manner utilizing solely the population-based cancer registry data is proposed in this paper. In total, eight scenarios concerning progress in incidence rates, survival rates, and the probability of an anti-tumour therapy administration are considered for the estimation of the number of potentially treated CRC patients. Based on the scenarios, the model predicts an increase in CRC prevalence ranging from 13% to 30% in comparison with the situation in 2008. The model also predicts that the number of colorectal cancer patients requiring active anti-tumour therapy in the Czech Republic in 2015 ranges from 10,074 to 11,440. Moreover, 3,485 to 4,469 patients will be treated for the metastatic disease, which accounts for more than one third of all CRC patients.

## Competing interests

The authors declare that they have no competing interests.

## Authors' contributions

TP and LD proposed the idea of the study. TP, OM and LD proposed the model, carried out the computations, and drafted the manuscript. JM and JK participated in the computations, model validation and data management. LS, JF, DF, and RV contributed to CNCR data validation and clinical interpretations. All authors read and approved the final manuscript.

## Pre-publication history

The pre-publication history for this paper can be accessed here:

http://www.biomedcentral.com/1471-2458/12/117/prepub
